# Thematic series: transcriptional regulation and disease

**DOI:** 10.1186/2045-3701-4-42

**Published:** 2014-08-19

**Authors:** Yi-Ching Wang

**Affiliations:** 1 Department of Pharmacology and Institute of Basic Medical Sciences, National Cheng Kung University, No.1, University Road, Tainan 70101, Taiwan

## 

In eukaryotic normal diploid cells, transcription must be accomplished in an efficient and faithful manner. The three nuclear RNA polymerases, transcription factors, chromatin regulators, and signaling pathways are among the key components that control gene expression. The molecular mechanisms by which these regulators control expression of individual genes have been studied extensively and are reviewed elsewhere [1,2]. Recently, various detective techniques and genome-wide profiling have shown that transcriptional deregulation including errors during RNA editing and epigenetic disorders gives rise to several significant human diseases including various cancers, neuron disorder, psychosis, and cardiovascular diseases [3,4].

In this issue of Cell & Bioscience, we have provided some evidences that deregulation of ribosomal gene (rDNA) transcription is a feature of cancer and other human disorders [5]. Critical issues with regard to how RNA editing crosstalks with other transcript-associated events to underpin regulated gene expression are discussed [6]. In addition, some new insights into the role of non-histone methylation and DNA demethylation in cancer progression are summarized [7]. We also highlight the current status of transcriptional and post-translational regulation of the DNA methyltransferase (DNMT) expression and activity with a focus on dysregulation involved in tumorigenesis [8].

Diesch *et al.* presented in their review entitled “Perturbations at the ribosomal genes loci are at the centre of cellular dysfunction and human disease” multiple aspects of rDNA functions. These functions not only include the relationship between RNA polymerase I (Pol I) transcription and proliferative property, but also the role of Pol I transcription in mediating heterochromatin structure and function, global gene expression and DNA damage response. Their review casts nucleolus as a central hub for coordination of stress responses. Notably, deregulation of Pol I transcription is required for malignant transformation and can be targeted with small molecule inhibitors to treat cancer [5].

Liu and associates summarized current technological advances in genomics methods in the study of functional impact of RNA editing and the adenosine deaminse acting on RNA (ADAR) family of enzymes on the regulation of gene expression in their review. In the past few years, research in the field of A-to-I RNA editing has been accelerated dramatically by the development of next generation sequencing (NGS) technology, which has expanded the landscape of RNA editome as well as our understanding of its regulation. Liu *et al*. reviewed the principles and techniques of the recent NGS-based studies that link RNA editing to various aspects of post-transcriptional RNA processing, alternative splicing, transcript stability and localization. Their review also pointed out the possibility that NGS data may be applied for further interrogation of the “RNA editing codes”, at which functional outcome could be explored in a global manner [6].

Baxter and colleagues systematically reviewed the alterations and mechanisms of DNA methylation and histone modifications in cancer. They described major findings on DNA demethylation resulting from replication-dependent passive dilution of 5-methylcytosine and active DNA demethylation catalyzed by Ten-eleven translocation (TET) family proteins. Baxter *et al*. emphasized on the transcriptional activity of HIF-α modulated by the histone methyltransferase G9a mediated methylation of non-histone proteins such as Reptin and Pontin in hypoxia. This review provides useful information for future therapeutic potentials by targeting the methylation of non-histone proteins under hypoxic conditions [7].

Lin and Wang evaluated in their review entitled “Dysregulated transcriptional and post-translational control of DNA methyltrasferases in cancer” the deregulation mechanisms of DNMTs. They focused on the transcriptional repression controls on *DNMT* genes by p53, RB and FOXO3a transcriptional regulators and corepressors. Interestingly, overexpressed MDM2 may induce *DNMT1, DNMT3A,* and *DNMT3B* expression by negative control over p53, RB and FOXO3a. In addition, the low expressions of miRNAs 29 s, 143, 148a and 152 are associated with overexpression of DNMTs in various cancers. In this review, several important post-translational modifications including acetylation and phosphorylation in the control of protein stability and activity of the DNMTs especially DNMT1 are discussed [8].

Due to the broad nature of transcriptional regulation and its misregulation in diseases, it is not our intention to cover all major aspects of this active research area in this single issue. However, we hope that these articles provide readers with insightful information of how alterations of Pol I transcription, RNA editing, non-histone methylation, and DNA methylation regulations may be used as disease biomarkers and for targeted therapies.
